# Facial expression recognition for emotion perception: A comprehensive science mapping

**DOI:** 10.1002/ibra.70010

**Published:** 2026-01-15

**Authors:** Hou‐Ming Kan, Li‐Ping Chen, Yu Zhang, Hao‐Yuan Hong, Ying‐Ying Qin, Yu‐Guo Cui, Yu‐Bo Mao, Yan‐Zhi Cheng, Zhe Lu, Hong‐Yan Ni, Xiao‐Tong Ding

**Affiliations:** ^1^ Faculty of Medicine Macau University of Science and Technology Macau SAR China; ^2^ Department of Pain Affiliated Hospital of Xuzhou Medical University Xuzhou China; ^3^ Department of Anesthesiology and Perioperative Medicine Suqian First Hospital Suqian China; ^4^ School of Psychological and Cognitive Sciences and Beijing Key Laboratory of Behavior and Mental Health Peking University Beijing China; ^5^ Key Laboratory of Machine Perception (Ministry of Education) Peking University Beijing China; ^6^ School of Nursing, Chinese Academy of Medical Sciences & Peking Union Medical College Beijing China

**Keywords:** artificial intelligence, bibliometric analysis, emotion perception, facial expression recognition, science mapping

## Abstract

Facial expression recognition (FER) has emerged as a pivotal interdisciplinary research domain that bridges computer science, psychology, neuroscience, and medicine. By mapping the FER scientific knowledge graph, this study aimed to explore the technological evolution and forecast future trends in this field. The study collected and cleaned the research on emotion perception in the Web of Science (WoS) database, and utilized the software CiteSpace (version 6.4R1) and R (BiblioShiny packages) software to create a scientific knowledge map. K‐means was used for cluster analysis, and then the latent Dirichlet allocation (LDA) was employed to extract popular topics from the text of each cluster. Uniform manifold approximation and projection (UMAP) was utilized to reduce high‐dimensional embeddings to a two‐dimensional space. From a regional perspective, research is mainly distributed in countries or regions such as North America, Western Europe, East Asia, India, and Australia. Research on facial emotion recognition has focused primarily on neuroscience, psychiatry, and psychology. With the rapid development of computer technology, the interdisciplinary intersection is becoming increasingly important as FER has shown strong potential in identifying rare and neurological diseases. Furthermore, the evolution of artificial intelligence (AI) has transformed facial expression feature extraction from manual methodologies to machine learning‐based approaches. The rapid development of computer algorithms and AI has greatly improved the accuracy and speed of facial emotion recognition. As a technology capable of detecting instantaneous emotional changes, FER holds promising prospects in fields such as neuroscience, emotion analysis, and pain assessment.

## Graphical abstract

Facial expression recognition (FER) has emerged as a pivotal interdisciplinary research domain, bridging computer science, psychology, neuroscience, and medicine. By mapping the FER scientific knowledge graph, the study aimed to explore the technological evolution and forecast future application trends in this field.

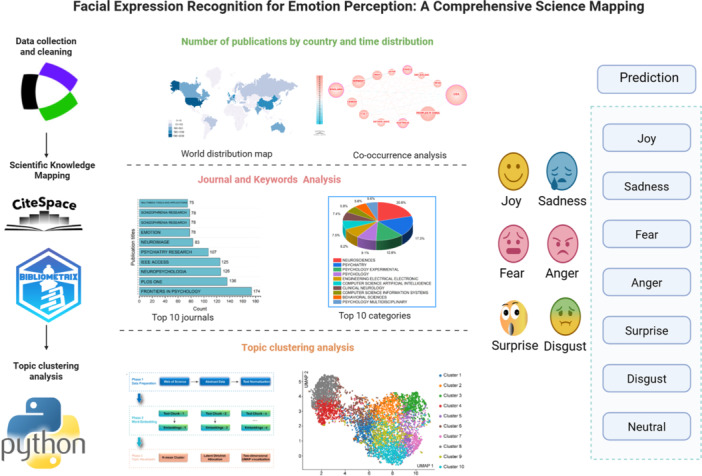

## 1 INTRODUCTION

Facial expressions represent the most immediate and biologically fundamental manifestation of human emotion and psychological state. Evolutionarily shaped to facilitate essential survival functions, emotions gradually evolve and exhibit distinct characteristics including sudden generation, transient effects, involuntary triggering, automatic evaluation, and response coherence.^1^ As early as 1970s, Ekman proposed the Facial Action Coding System (FACS), which links the movement of facial muscles with emotions.^2^ Through analysis of facial muscle movement, facial expressions are divided into multiple action units (AUs), and six basic emotions are defined. Moreover, emotions are universal, but people's facial expressions in different cultures, races, and regions are highly consistent.^3^ FACS theory provides a standardized tool for psychological emotion recognition and a theoretical basis for applying computer vision in expression recognition. Advances in artificial intelligence (AI) technology have significantly increased the efficiency and precision of emotion recognition. As a result, facial expression recognition (FER) has emerged as an important interdisciplinary research domain spanning psychology, computer science, neuroscience, and medicine. With the iteration of deep learning technology and the availability of diverse large‐scale datasets, FER research has experienced explosive growth, accompanied by increasing diversification in research topics, methodologies, and technical approaches. FER plays an important role in evaluating emotional changes and therapeutic efficacy in individuals with mental disorders. Under these circumstances, adopting a scientific knowledge mapping system to analyze the knowledge structure, identify research hotspots, and detect evolution trends will not only help elucidate the developmental trajectory of FER but also address the challenges of current development and provide a potential direction for FER.

With the rapid development of AI and deep learning, FER models have been rapidly applied to emotion recognition in recent years. By training a small number of datasets, more accurate emotion recognition can be obtained, expanding the application of FER in clinical medicine. The combinations of FER with electroencephalography (EEG), eye tracking, and other technologies for multimodal emotion recognition and the development of brain computer interfaces promote traditional emotion recognition and provide possibilities for exploring more complex advanced brain functions. The previous reviews mainly focused on the differences between algorithm models, but overlooked the exploration of the potential application of FER in different fields. This study used science mapping analysis to systematically review the current application status and development trends of facial recognition technology in emotion recognition.

## 2 METHODS

### 2.1 Overview of the research process and analytical methods

This study determined FER technology as the research topic, with a focus on analyzing past research priorities and future research directions. Subsequently, data extraction and cleaning were carried out, followed by using scientometric analysis. Scientometrics primarily examines research trends through citation analysis, co‐occurrence, and network science to identify historical research priorities. To explore the potential future research directions in FER, the study additionally adopted in‐depth topic clustering analysis of the abstract to infer emerging scientific frontiers. The analytical results yield a holistic perspective on the evolution of FER technology and propose applications for diverse potential populations.

### 2.2 Data collection and cleaning

The study used “facial recognition” and “emotion” as keywords for retrieval. After the search terms were determined, we systematically searched in the Web of Science (WoS) Core database and completed the search on April 10, 2025. The principles of data collection and cleaning are as follows: (1) the search strategy is presented in Table 1; (2) only peer‐reviewed articles were included; conference proceedings, letters/correspondence, revisions, and retracted papers were excluded; (3) the publication date was unrestricted; and (4) the publication language is defined as English. After using NoteExpress (Aegean, Beijing) for deduplication, two researchers independently screened the articles by reading the titles and abstracts, and the disputed literature was adjudicated by a third researcher.

**Table 1:** Search terms and strategies.

*Database*	
Science Citation Index Expanded (SCI‐EXPANDED), Social Sciences Citation Index (SSCI), Emerging Sources Citation Index (ESCI)	
*Search terms and strategies*	
Query #1 31,304 records	“Face recognition” (Topic) or “Facial Identity Recognition” (Topic) or “Face Processing” (Topic) or “Face Perception” (Topic) or “Facial Emotion Recognition” (Topic) or “Face Emotion Recognition” (Topic) or “Face Emotion Processing” (Topic) or “Facial Expression Recognition” (Topic) or “Facial Expression Recognition” (Topic)
Query #2 1,684,899 records	“Emotion” (Topic) or “Mood” (Topic) or “Feeling” (Topic) or “Anger” (Topic) or “Rage” (Topic) or “Anxiety” (Topic) or “Catastrophization” (Topic) or “Apathy” (Topic) or “Bereavement” (Topic) or “Grief” (Topic) or “Boredom” (Topic) or “Courage” (Topic) or “Depression” (Topic) or “Disgust” (Topic) or “Emotional Exhaustion” (Topic) or “Emotional Regulation” (Topic) or “Euphoria” (Topic) or “Fear” (Topic) or “Panic” (Topic) or “Forgiveness” (Topic) or “Frustration” (Topic) or “Guilt” (Topic) or “Shame” (Topic) or “Happiness” (Topic) or “Hate” (Topic) or “Hope” (Topic) or “Hostility” (Topic) or “Jealousy” (Topic) or “Loneliness” (Topic) or “Love” (Topic) or “Pleasure” (Topic) or “Sadness” (Topic) or “Psychological Distress” (Topic)
Query #3 7119 records	#1 AND #2
*Date of literature retrieval*	
April 10, 2025	

### 2.3 Scientific knowledge mapping

The study utilized Microsoft Excel 2021 (Microsoft Corporation, USA) to visualize the world distribution of literature density. Column charts, sector charts, and volcano charts for quantity statistics were generated via OriginPro 2024 (OriginLab Corporation, USA). In this study, we used CiteSpace software (version 6.4.R1) to conduct co‐occurrence analysis of countries and keywords, as well as keyword burst analysis.^4^ Slice length was set to 1 and g‐index to *k* = 25 to understand the important research achievements and development trends in this field. The Biblioshiny package in R was used to conduct trend topic analysis on keywords to understand hot topics in the field of FER research.^5^ The node size represents the frequency of occurrence, and the line length represents the time span. After completing data collection and cleaning, we completed the production of the science mapping on April 26, 2025.

### 2.4 Topic clustering analysis

The abstracts of the included studies were exported to Excel. Textual information was transformed into vector representations via the Word2Vec model in Python. The K‐means algorithm is used to aggregate document vectors into K clusters and identify document groups with similar semantic content. To maintain a balance between granularity and interpretability, 10 clusters were selected for manual interpretation and labeling based on the silhouette score. The representative topic words from each cluster were extracted via latent Dirichlet allocation (LDA), and the core topic content of each cluster was identified. Finally, high‐dimensional embeddings were reduced to a two‐dimensional space via uniform manifold approximation and projection (UMAP), with clusters color‐coded and topic labels annotated.^6^ The code for topic clustering analysis can be found on GitHub at https://github.com/jdheu34/Topic-clustering-analysis.git.

## 3 RESULTS

### 3.1 Global publication distribution and temporal trends

**Figure 1:**
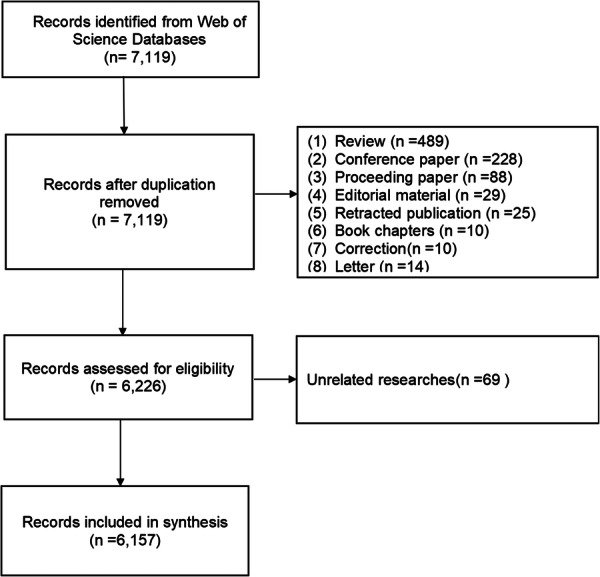
The flowchart of literature inclusion and exclusion.

**Figure 2:**
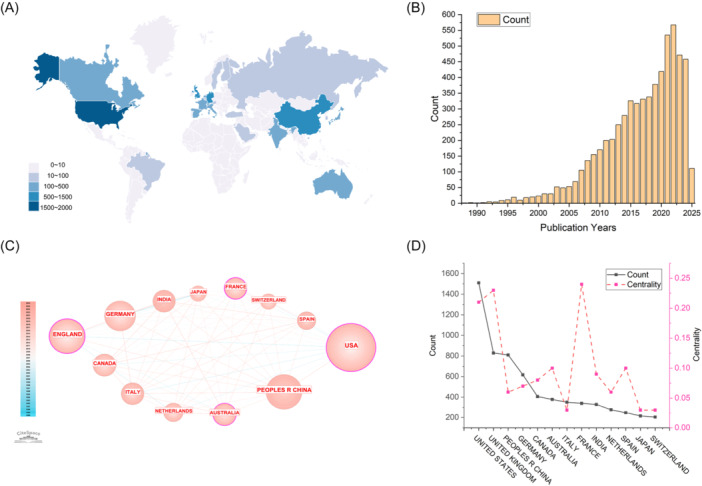
Global publication distribution and temporal trends. (A) Global distribution of research publications. (B) Temporal trend of publications. (C) Co‐occurrence analysis of the main publishing countries or regions. (D) Publication output and network centrality of leading countries/regions.

A total of 7119 records were obtained through a preliminary search. After removing 893 nonartistic records and 69 records unrelated to the topic, a total of 6157 records were included (Figure 1). To understand the number of papers published in the field of FER in different countries or regions, a global academic output distribution heatmap was created. The darker the color is, the greater the number of articles published in the region. Geographically, research output was primarily concentrated in countries or regions such as North America, Western Europe, East Asia, India, and Australia (Figure 2A). The number of publications has increased yearly since the 1990s, peaking in 2022, followed by a slight decline in the following 2 years (Figure 2B). Nodes with high centrality represent their role as bridges or hubs in the knowledge structure. The centrality calculation result is a normalized value, usually between 0 and 1. Nodes with centrality ≥ 0.1 are usually considered key hub nodes. China ranks third in the world in terms of the number of published articles (*n* = 810), which is lower than that reported by the United States (*n* = 1510) and the United Kingdom (*n* = 829). However, China's centrality is only 0.06, which is far lower than that of the United States (0.21), the United Kingdom (0.23), and France (0.24) (Figure 2C,D). Although China has a relatively high number of published papers worldwide, there is a relative lack of high‐quality and influential papers.

### 3.2 Journal and science category analysis

**Figure 3:**
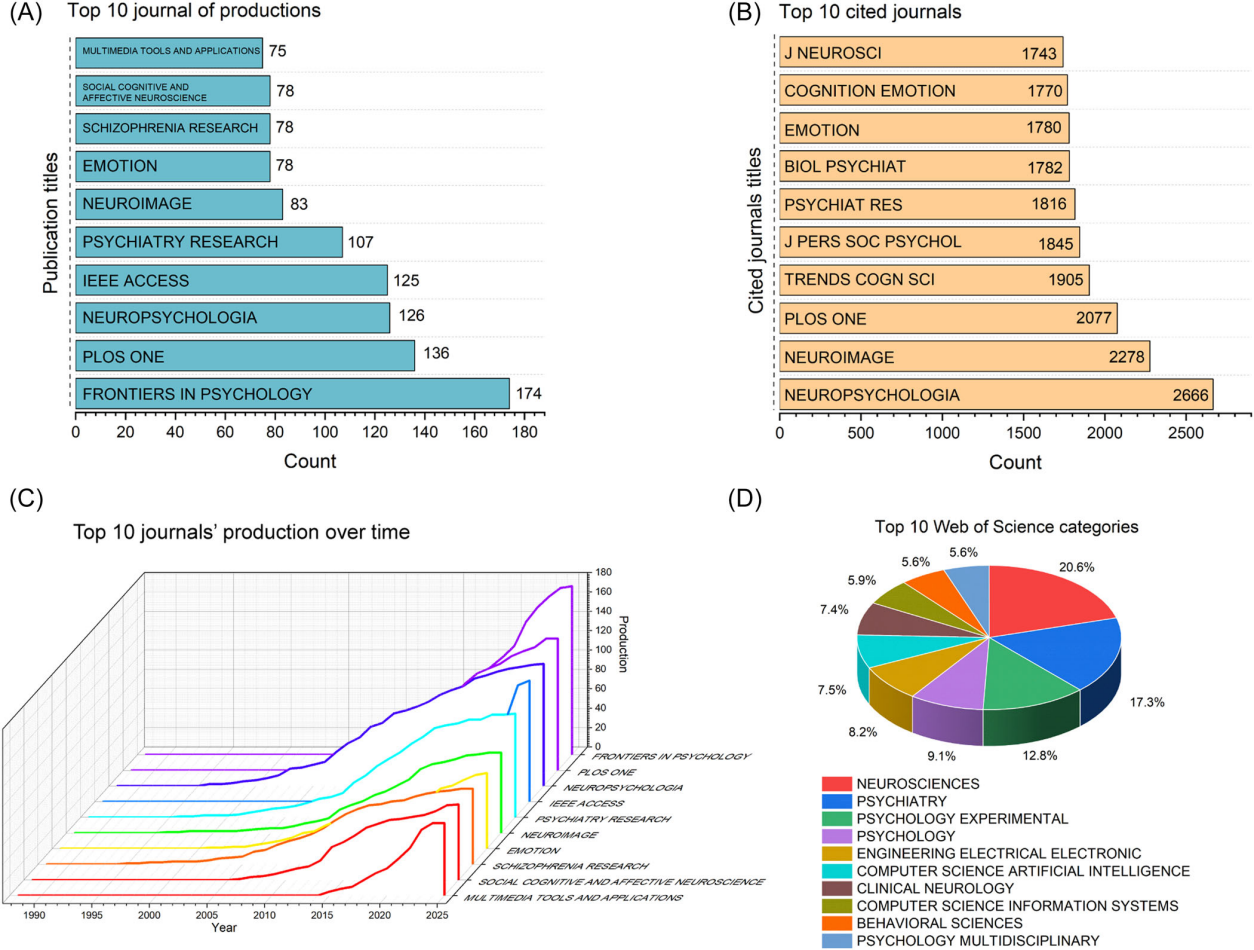
Journal and science category analysis. (A) Top 10 most prolific journals. (B) Top 10 most cited journals. (C) Publication trends of the leading journals. (D) Top 10 most frequent Web of Science categories.

The three journals with the highest publication counts were *Frontiers in Psychology* (*n* = 174), *PLOS ONE* (*n* = 136), and *Neuropsychologia* (*n* = 126) (Figure 3A). The top three cited journals were *Neuropsychologia* (*n* = 2666), *Neuroimage* (*n* = 2278), and *PLOS ONE* (*n* = 2077) (Figure 3B). *IEEE Access* has experienced particularly rapid growth in publication volume in recent years, which may be attributed to the interdisciplinary development of computer science and medicine (Figure 3C). Analysis of the WoS categories revealed *Neurosciences*, *Psychiatry*, and *Psychology Experimental* as the top three research fields (Figure 3D).

### 3.3 Keyword citation bursts and co‐occurrence analysis

**Figure 4:**
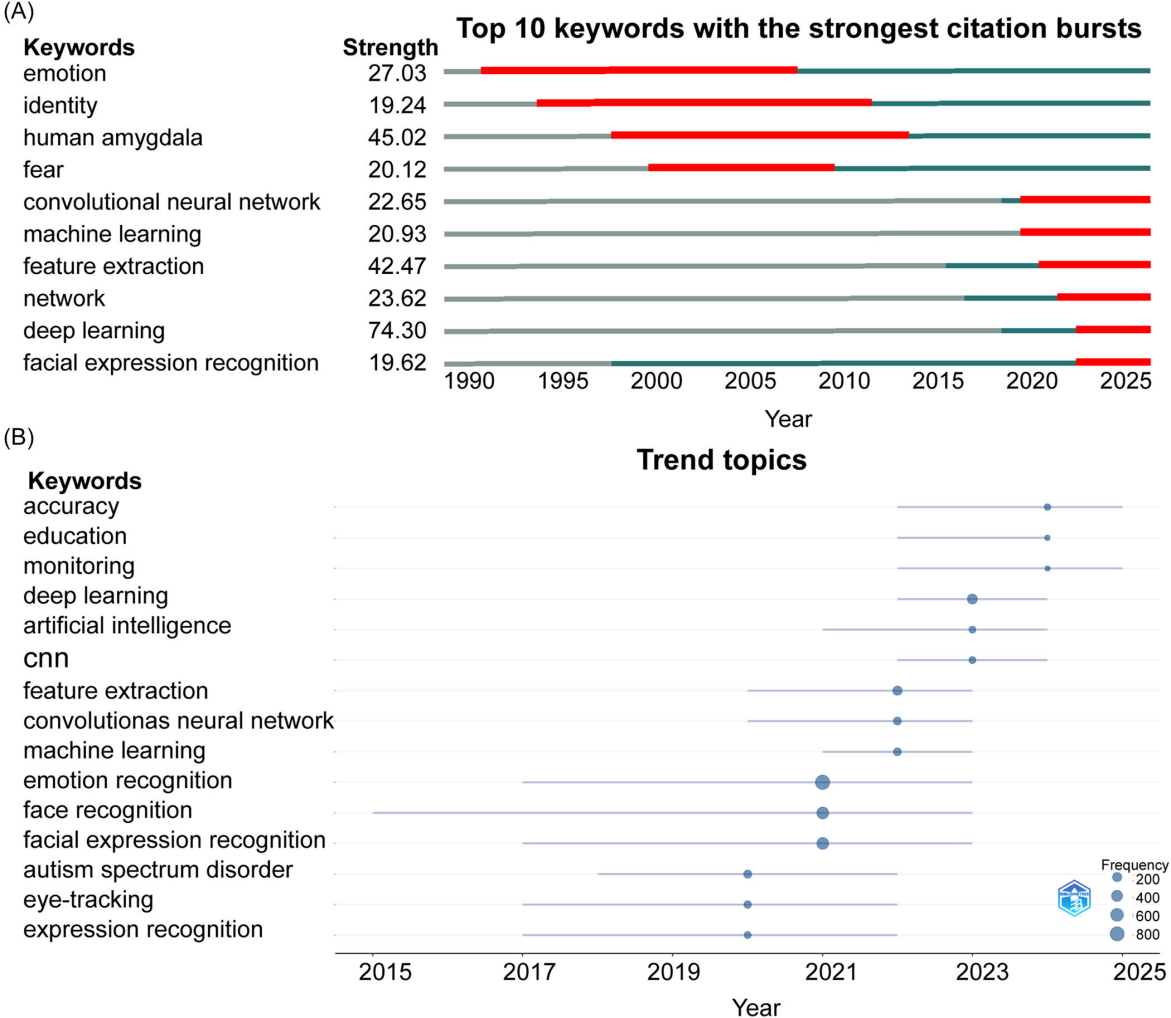
Keyword citation bursts analysis. (A) Top 10 keywords with the strongest citation bursts. (B) Trend topics of keywords in the past decade.

**Figure 5:**
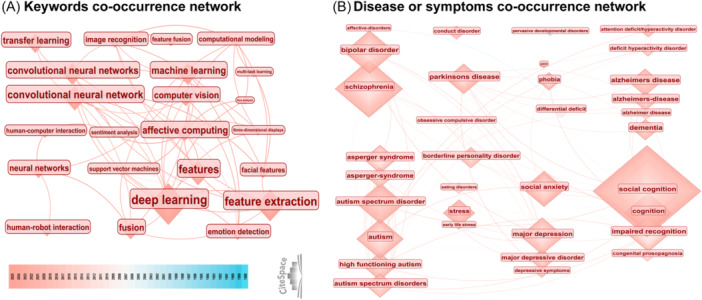
Keyword co‐occurrence analysis. (A) Co‐occurrence network of technology‐focused keywords. (B) Co‐occurrence network of disease/symptom‐focused keywords.

The timeline view of keyword citation bursts reveals the evolution of research hotspots in this field. Early research primarily focused on neuroscience, particularly the neural mechanisms of the amygdala in fear emotions. Subsequent advances in machine learning, deep learning, and neural networks have provided technical support for emotion recognition technology. At present, the feature fusion optimization of emotion recognition is based on convolutional neural networks (CNNs) and cross‐modal emotion data (e.g., eye tracking), marking a transition from basic neuroscience to AI‐driven emotion computing (Figure 4A,B). The co‐occurrence network of keywords reveals the core research directions in emotion recognition, including deep learning, computer vision, feature fusion, transfer learning, and human–computer interaction, demonstrating the trend of multimodal technology fusion, especially the cross‐application of feature fusion and emotion recognition (Figure 5A). Emotion recognition technology has been predominantly applied to four clinical categories: emotional disorders (e.g., depression, bipolar disorder), neurodevelopmental disorders (e.g., Asperger syndrome, autism spectrum disorder), neurodegenerative diseases (e.g., Alzheimer's disease, Parkinson's disease), and behavioral and emotional disorders (e.g., conduct disorder, phobia) (Figure 5B).

### 3.4 Topic modeling analysis

**Figure 6:**
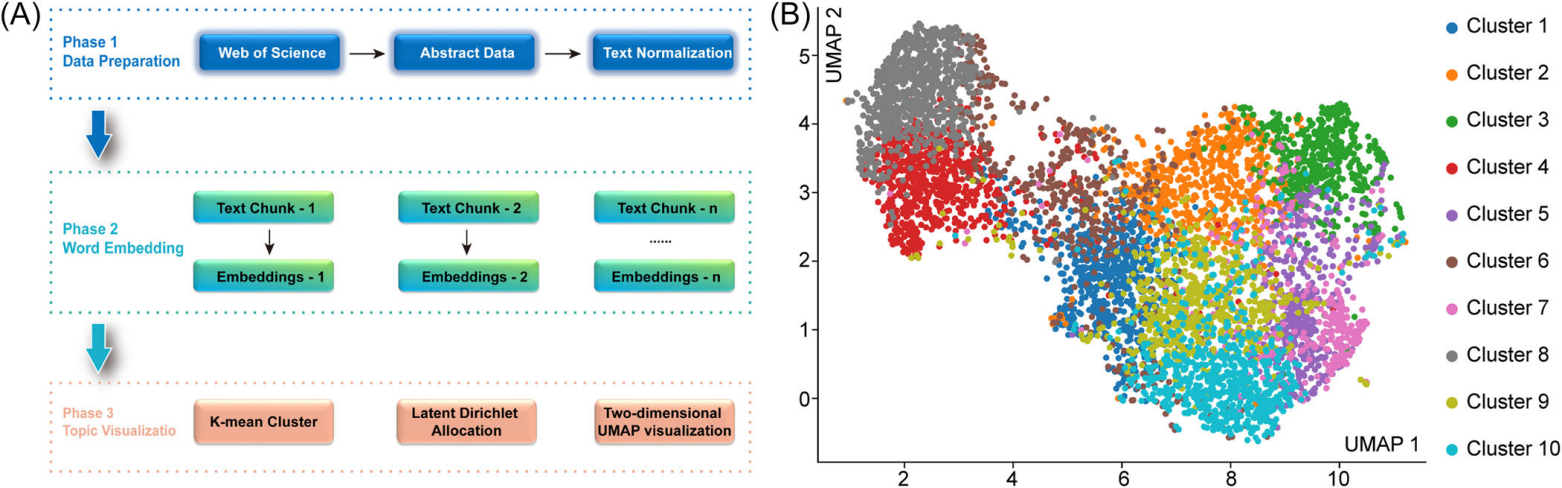
Two‐dimensional visualization of topic clusters. (A) Topic modeling workflow. (B) Topic clusters visualized by uniform manifold approximation and projection (UMAP).

Ten topics were extracted after clustering via 2‐dimensional UMAP (Figure 6). Each cluster corresponds to a specific set of keywords detailed in Table 2. The results revealed that algorithm development research, such as deep learning and feature engineering, has become the most active field.

**Table 2:** Topic clusters and keywords identified in topic modeling analysis.

Part	Cluster	Keywords	Items
Part 1 Computational science and technology development	Cluster 4 Artificial intelligence models and algorithms	Emotion, recognition, facial, expression, face, system, **method**, **feature**, **model**, **learning**	763
	Cluster 8 Computer vision methods	Facial, expression, feature, recognition, emotion, face, **proposed**, **method**, **model**, **image**	834
Part 2 Social cognition and clinical application	Cluster 5 Clinical population study	Emotion, recognition, facial, study, face, emotional, processing, **control**, **group**, **patient**	500
	Cluster 7 Social cognitive impairment	Recognition, emotion, cognition, study, facial, patient, face, **disorder**, **cognitive**, **social**	346
	Cluster 9 Research on developmental groups	Face, recognition, study, facial, emotion, group, processing, emotional, **patient**, **child**	644
	Cluster 10 Clinical evaluation application	Emotion, recognition, facial, expression, face, emotional, study, patient, **group**, **control**	674
Part 3 Exploration of basic neural mechanisms	Cluster 2 Normal emotional processing	Face, processing, expression, emotional, facial, emotion, study, **neutral**, **stimulus**, **response**	665
	Cluster 3 Neurological basis and patients	Face, processing, emotional, patient, emotion, facial, study, **brain**, **neural**, **amygdala**	520
Part 4 Integrated and core methodology	Cluster 1 Mechanisms for FER	Face, expression, emotion, facial, recognition, participant, emotional, study, processing, **effect**	749
	Cluster 6 Outcome analysis	Facial, expression, face, emotion, recognition, emotional, processing, **study**, result, participant	452

*Note*: Bold text indicates keywords that are distinctly characteristic of the corresponding cluster and clearly distinguish it from other clusters.

These 10 clusters clearly outline the four core pillars of FER research. The first part is the development of computational science and technology, which includes Cluster 4 and Cluster 8. Cluster 4 represents AI models and algorithms, with keywords such as model, learning, method, and feature indicating that its core focus is on developing new algorithms and feature extraction techniques. Cluster 8 represents computer vision methods, with keywords such as proposed, method, model, and image emphasizing the proposal of new methods and systems to solve specific engineering problems. The second part concerns social cognition and clinical applications, and includes Cluster 5, Cluster 7, Cluster 9, and Cluster 10. Cluster 5 represents a clinical population study that compares the abnormal facial expression processing of patients with specific diseases with that of the control group. Cluster 7 represents social cognitive impairment, focusing on higher level social and cognitive functions, and how the disorder affects these functions. Cluster 9 represents the study of developmental groups, and the keyword “child” indicates that its focus is on the development process of emotional abilities and specific obstacles in the child population. Cluster 10 represents clinical evaluation applications, focusing on using emotion recognition as a tool for group comparison and clinical control assessment. The third part explores basic neural mechanisms, including Cluster 2 and Cluster 3. Cluster 2 represents normal emotional processing, which uses stimuli to study the response of healthy brains, with a focus on the differences in processing neutral and emotional faces. Cluster 3 represents the relationship between the neural basis and the patients, with keywords amygdala, brain, and neural, which directly indicate their neuroscience attributes. By studying patients, specific brain regions involved in emotional processing can be located. The fourth part is the synthesis and core methodology, which includes Cluster 1 and Cluster 6. These two clusters constitute the cornerstone of the field, representing classic psychological experimental research and providing basic paradigms and theoretical support for other branches.

## 4 DISCUSSION

Human emotion represents a complex psychophysiological phenomenon. Ekman's foundational work in the 1970s proposed a definition of emotion that underpins modern emotion recognition research.^2^ Discrete emotions and dimensional emotions are currently the two mainstream emotion models, which offer complementary perspectives on emotional phenomena and inform the development of facial expression databases.^7^, ^8^ FER‐related research began in the 1990s, and the number of related studies has gradually increased, indicating that FER is a promising field. FER research has focused mainly on North America, China, Western Europe, India, and Australia. The United States, England, and France have high centrality, indicating that these countries play an important role in this field. In terms of publishing journals and fields, FER is highly regarded not only in the medical field but also in the engineering and computer fields.

### 4.1 Traditional FER to deep learning and AI mode

Facial expressions represent the most intuitive visual signals for emotional communication, serving as crucial nonverbal indicators of human intentions. Ekman and Friesen's FACS remains the gold standard for FER.^9^ However, technological limitations and imaging conditions often constrain the emotional information extracted from facial expressions. In the late 1990s, facial emotion recognition technology achieved several key breakthroughs in algorithm innovation, dataset construction, and interdisciplinary integration. Technological evolution has shifted facial expression feature extraction from manual methods to machine learning approaches. The analysis of keyword burst intensity revealed that deep learning (strength = 74.30) and feature extraction (strength = 42.47) have very high intensities, indicating that deep learning is now the driving force of the FER core and that feature extraction has always been the core problem in this field (Figure 4A). Gabor filters and elastic graph matching (EGM) form a sparse graph structure of facial key‐points and subtle textures, improving the accuracy of dynamic expression and micro‐expression analysis.^10^ The research trends and durations indicate that deep learning, AI, machine learning, and other technological methods have become mainstream in recent years, and FER models have learned how to recognize expressions through massive amounts of data (Figures 4B and 5A). The Japanese Female Facial Expression (JAFFE) database, developed by Lyons, provides annotated datasets that facilitate algorithm comparison and validation.^11^ Early facial recognition technology relied primarily on static image features, including Gabor features,^12^ local binary pattern (LBP) features,^13^ and principal component analysis (PCA) features.^14^ Dynamic facial expression recognition (DFER) can obtain temporal emotional changes from video or image sequences, which is a part of the development of intelligent human–computer interaction systems. Maruthapillai and Murugappan achieved real‐time recognition of facial expressions via an optical flow algorithm for facial landmark tracking.^15^, ^16^ Recent AI advancements have enabled deep learning methods for complex scenes and large‐scale data analysis. Notably, deep learning methods include deep convolutional neural networks (DCNNs), recurrent neural networks (RNNs), generative adversarial networks (GANs), transformers, and comparative language image pretraining (CLIP).^16^ Karnati et al. proposed FER‐net based on a CNN, which effectively distinguishes facial expressions using a softmax classifier.^17^ To address the issues of overfitting and intra‐class facial appearance variations in traditional CNNs, they proposed a texture‐based feature‐level ensemble parallel network (FLEPNet) on the original model to improve the performance of FER systems.^18^ With the development of AI technology, transfer learning models are becoming a new paradigm in the field of FER and a cutting‐edge exploration direction in this field (Figure 5A).

### 4.2 Pain assessment

Pain is a subjective feeling with both sensory and emotional dimensions.^19^ Pain assessment relies mainly on self‐assessment scales, which lack objective indicators. This poses significant challenges in patients unable to reliably self‐report pain, such as infants, critically ill or intubated patients, and individuals with dementia or severe communication impairments. To address these challenges, using machine learning models to analyze facial expressions can be used to evaluate postoperative pain and patients who need rescue analgesia.^20^ Some models have also been developed for pain assessment in neonates and patients with dementia, such as the InceptionV3,^21^ PainChek® Adult,^22^ and FaceReader9^23^ models, which have achieved satisfactory accuracy and F1 scores. The Pose invariant Occlusion robust Pain Assessment (POPA) framework can be used to assess pain in newborns based on their facial features, which is crucial for improving pain management in newborns.^24^ FER models applied to pain assessment can be found in Table 3.

**Table 3:** Summary of common facial expression recognition models on pain and disease evaluation.

Models	Experimental population	Reported performance metrics
AU formula model^20^	Postoperative pain	AU17 and AU20 were associated with severe pain, but the predictive performance of head posture and facial landmarks (AUROC 0.85–0.96) was better than that of action units. The comprehensive model that integrates eye movement, eye landmarks, and head posture has the best prediction performance (AUROC 0.90).
InceptionV3^21^	Pain assessment in adults with cerebral palsy	The InceptionV3 model achieved an accuracy of 62.67% and an F1 score of 61.12% on the CP‐PAIN dataset to recognize pain.
PainChek®^22^	Pain assessment in Aboriginal aged care residents with cognitive impairment	After statistical adjustment, pain scores generated by the PainChek® Adult app's automated facial analysis were comparable between the Aboriginal residents and non‐Aboriginal residents.
FaceReader9^23^	Neonatal procedural pain	The artificial intelligence software FaceReader 9 was used to analyze facial action units, and its measured arousal and valence indicators were highly correlated with expert pain scores (*r* = 0.84).
POPA^24^	Newborn	A pain dataset containing 1091 newborns, used for the POPA framework facial expression recognition task.
Face2Gene^25^	Children with Kabuki Syndrome	Among the 99 Kabuki Syndrome patients who provided facial photos, after removing 14 patients with poor image quality, Face2Gene software was able to accurately diagnose the remaining 85 patients with a success rate of 100%.
AI‐FR^26^	Turner Syndrome	In a real‐world cohort of 218 patients (29 Turner Syndrome and 189 controls), the AI‐FR system achieved an accuracy of 89.0% and an AUROC of 0.820.
Face++^27^	Parkinson disease	The application of a long short‐term model neural network can achieve an accuracy 86% and F1 score of 75%. Furthermore, using a support vector machine algorithm to detect facial micro‐expressions can achieve an F1 score of 99%.
3D ResNet‐50^28^	Myasthenia gravis	A deep learning model trained on facial expression video data was used to identify the severity of a patient's condition.
CmdVIT^29^	Complex mental disorders	CmdVIT has achieved satisfactory accuracy and F1 scores in various psychiatric datasets. Specifically, CmdVIT recognizes best in neutral expression, intermediate in pleasure and surprise expression, and poorly in anger and fear recognition.
IDenseNet‐RCAformer^30^	Autism spectrum disorder	This former achieved 98.95% FER accuracy, 97.70% optimal specificity, and an F1 score of 96.84%, which was applied to effectively recognize certain facial expressions in patients with autism.
RetinaFace^31^	Employee	The model achieved high accuracy in emotion classification, with VGG16 outperforming MobileNet and ResNet50 in key metrics such as accuracy, precision, and recall.

Abbreviations: AI‐FR, artificial intelligence‐based facial recognition; AU, action unit; AUROC, area under the receiver operating characteristic curve; FER, facial expression recognition; POPA, Pose‐invariant Occlusion‐robust Pain Assessment.

### 4.3 Disease evaluation

FER also shows strong potential in identifying rare diseases^25^, ^26^ and neurological diseases.^32^ Jin et al. used Face++ to extract facial expression features and then applied deep learning algorithms to diagnose Parkinson's disease, achieving an accuracy of 86% and an F1 score of 75%. In addition, the support vector machine algorithm was used to process facial micro‐expressions, and the F1 value increased to 99%.^27^ FER can also be used to assess the severity of diseases. Patients with myasthenia gravis (MG) may have involvement of extraocular muscles, and the use of 3D ResNet‐50 can accurately classify the severity of the disease.^28^ FER can quickly identify the emotional state of healthy individuals or patients with mental disorders. At present, several models have been developed for psychologically assisted diagnosis. For example, the Vision Transformer FER (CmdVIT) is used to assess the emotional state of patients with mental disorders,^29^ the IDenseNet‐RCA is used to assess autism spectrum disorders,^30^ and the RetinaFace model to assess the emotional state of employees.^31^ More details of the FER model for disease assessment can be found in Table 3.

### 4.4 AI in traditional Chinese medicine

Traditional Chinese medicine (TCM) practitioners believe that specific areas of the face contain the health status of Zang Fu organs. Facial assessment has always been an important component of TCM. The research on facial automated TCM diagnosis based on AI is exploring and developing. The use of deep neural network technology to analyze pulse wave signals in facial videos can be used to assist in blood pressure prediction, providing a convenient method for the management of cardiovascular diseases.^33^ The use of emotional computing and AI in the diagnosis and treatment process of TCM can establish emotional models tailored to the patient's mood. This utilization of the five‐tone intelligent diagnosis system greatly improves the accuracy of diagnosis and treatment.^34^

### 4.5 Humanoid robot

In recent years, humanoid robots have made rapid progress in both mobility and coordination. With the development of AI and flexible electronic devices, humanoid robots have gradually achieved basic facial expressions and preliminary anthropomorphism.^35^ Humanoid robots with diverse facial expressions have received more attention in human–computer interaction, especially in psychological testing and promoting mental health. At present, there is still a lack of quantitative evaluation methods for the facial expressions of humanoid robots.^36^ Developing FER specifically for humanoid robots is beneficial for providing emotional personification of robots and bridging the gap between humanoid robots and humans.^37^

### 4.6 Multimodal emotion recognition

**Figure 7:**
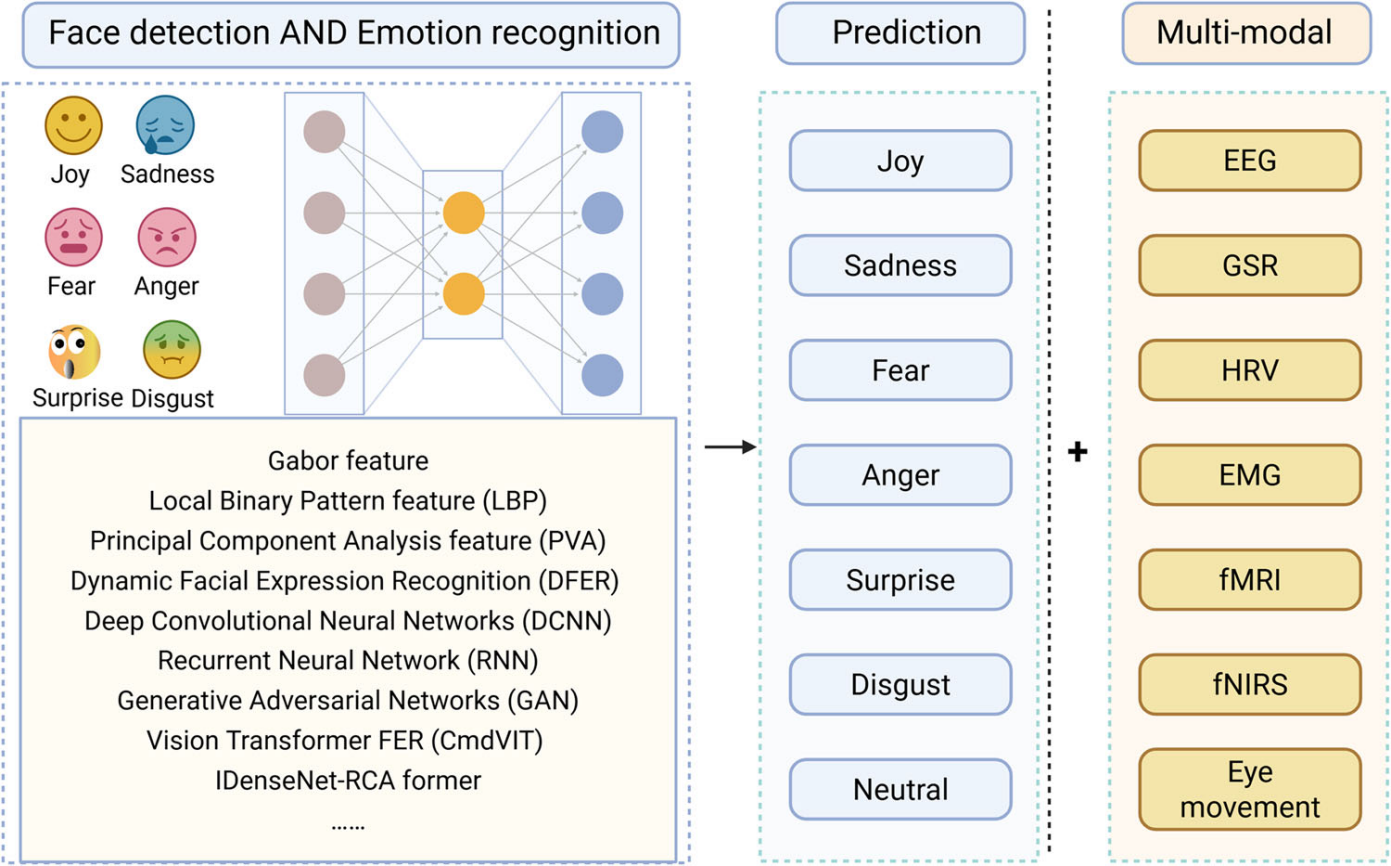
Technical framework for facial emotion perception and multimodal integration. EEG, electroencephalography; EMG, electromyography; fMRI, functional magnetic resonance imaging; fNIRS, functional near‐infrared spectroscopy; GSR, galvanic skin response; HRV, heart rate variability.

In emotion recognition tasks, single FER has limitations due to the subjectivity and fraud of facial expressions. Integrating emotional signals with objective physiological or neuroimaging data can significantly improve recognition performance. The current technologies that can perform multimodal analysis^38^, ^39^, ^40^, ^41^ with FER include EEG, galvanic skin response (GSR), heart rate variability (HRV), electromyography (EMG), eye movement, functional magnetic resonance imaging (fMRI), and functional near‐infrared spectroscopy (fNIRS) (Figure 7). Multimodal emotion recognition integrates multidimensional data, significantly improving the accuracy of emotion analysis, and provides the possibility of using a brain–computer interface (BCI) for pain management, psychological intervention, and emotion detection in the future. The Brain Machine Generative Adversarial Networks (BM‐GAN) were used to learn cognitive features from EEG signals triggered by facial emotional images. After training, the model achieved an accuracy of 96.6% in recognizing facial emotional images.^42^ The introduction of BCI is expected to transform open‐loop multimodal recognition into a closed‐loop intervention paradigm. A personalized recognition system based on multimodal emotions that uses advanced cross‐modal fusion algorithms, such as attention mechanisms and transformers, to balance the reliability of different signals and decode emotions and pain states. After emotional or pain signals are recognized, the system automatically provides neural regulation or other interventions, forming a real‐time and automatic closed loop. However, it must be noted that this deep integration technology also brings serious ethical challenges, and it is necessary to establish relevant ethical and regulatory frameworks simultaneously, which is itself an important future research direction.^43^

### 4.7 Ethical and clinical considerations for FER

Given that facial recognition is a sensitive biometric information about individuals, strict ethical review and regulation must be followed when using facial recognition in clinical practice. For special situations such as mental illness, children, dementia, etc., patients should be the center and communicate with themselves and their guardians with full respect. When collecting facial data, attention should be paid to data security to prevent data leakage. Algorithmic FER also leads to biased decisions, necessitating that medical staff be fully involved in the decision‐making process.

### 4.8 Advantages and limitations

There are few bibliometric studies on FER that can be retrieved. Girdhar et al. conducted research on the identification of common biomedical features, which included facial recognition, fingerprint and palm print recognition, iris and retina scanning, speech and speech analysis, gait recognition, and DNA‐based recognition. However, this study did not focus on facial expressions.^44^ Ahmad et al. conducted a bibliometric analysis on facial micro‐expressions and deep learning. However, this study was limited to facial micro‐expressions and did not explore the potential applications of FER.^45^ Our study thoroughly investigated the common models and potential applications of FER for emotions through science mapping methods. Its interdisciplinary approach involves new research avenues in neuroscience, psychology, psychiatry, and clinical medicine.

However, this study has several limitations. First, to avoid information loss caused by format conversion, we only included the WoS database to ensure data reliability, but this may also result in the omission of relevant studies from other sources. In the future, the database scope can be expanded by transcoding citation formats. Second, our study did not conduct subgroup analysis on different fields, so we were unable to obtain specific characteristics of FER research in different fields. In the future, bibliometric analysis can be conducted in specific fields such as psychology, psychiatry, and pain assessment to explore research directions and hotspots that are more suitable for this field.

## 5 CONCLUSION

This scientific knowledge mapping study depicts the rapid evolution of the field of facial expression analysis, shifting from classical computer vision methods to complex multimodal AI systems. With the development of AI and deep learning, the field of FER research has expanded to include the monitoring of neurological diseases, assessment of mental health, and objective pain assessment for noncommunicating patients. However, the development of this field faces challenges because of its reliance on laboratory control data. Therefore, future work must prioritize the development of robust, interpretable multimodal AI models based on diverse real‐world data to ensure their clinical translational and ethical applications. This study provides a comprehensive roadmap that can guide researchers in filling key gaps and promoting the development of next‐generation emotion computing technology.

## AUTHOR CONTRIBUTIONS

Hou‐Ming Kan and Li‐Ping Chen designed the study, performed data analysis, and wrote and drafted the article. Yu Zhang, Hao‐Yuan Hong, and Ying‐Ying Qin contributed to data curation, and writing and drafting the article. Yu‐Guo Cui, Yu‐Bo Mao, Yan‐Zhi Cheng, and Zhe Lu critically revised the article. Hong‐Yan Ni and Xiao‐Tong Ding provided software operation guidance, edited the manuscript, and revised and approved the final version of the article.

## CONFLICT OF INTEREST STATEMENT

The authors declare no conflicts of interest.

## ETHICS STATEMENT

This study involves no primary human participants, relying solely on secondary data analysis from publicly available bibliometric records.

## Data Availability

Data sharing is not applicable to this article as no new data were created or analyzed in this study.

## References

[1] Ekman P . An argument for basic emotions. Cogn Emot. 1992;6(3‐4):169‐200. 10.1080/02699939208411068

[2] Ekman P . Universals and cultural differences in facial expressions of emotion. Nebr Symp Motiv. 1971;19:207‐283.

[3] Brooks JA , Kim L , Opara M , et al. Deep learning reveals what facial expressions mean to people in different cultures. iScience. 2024;27(3):109175. 10.1016/j.isci.2024.109175 38433918 PMC10906517

[4] Chen C , Song M . Visualizing a field of research: a methodology of systematic scientometric reviews. PLoS One. 2019;14(10):e0223994. 10.1371/journal.pone.0223994 31671124 PMC6822756

[5] Aria M , Cuccurullo C . bibliometrix: an R‐Tool for comprehensive science mapping analysis. J Informetr. 2017;11(4):959‐975. 10.1016/j.joi.2017.08.007

[6] Healy J , McInnes L . Uniform manifold approximation and projection. Nat Rev Methods Primers. 2024;4(4):82. 10.1038/s43586-024-00363-x

[7] Fujimura T , Umemura H . Development and validation of a facial expression database based on the dimensional and categorical model of emotions. Cogn Emot. 2018;32(8):1663‐1670. 10.1080/02699931.2017.1419936 29334821

[8] Takehara T , Ochiai F , Suzuki N . A small‐world network model of facial emotion recognition. Q J Exp Psychol. 2016;69(8):1508‐1529. 10.1080/17470218.2015.1086393 26315136

[9] Caltagirone C , Ekman P , Friesen W , et al. Posed emotional expression in unilateral brain damaged patients. Cortex. 1989;25(4):653‐663. 10.1016/s0010-9452(89)80025-5 2612182

[10] Duc B , Fischer S , Bigun J . Face authentication with Gabor information on deformable graphs. IEEE Trans Image Process. 1999;8(4):504‐516. 10.1109/83.753738 18262894

[11] Lyons MJ , Akamatsu S , Kamachi MG , Gyoba J . Coding facial expressions with Gabor wavelets. In: Proceedings Third IEEE International Conference on Automatic Face and Gesture Recognition, Nara, Japan, 1998:200‐205. 10.1109/AFGR.1998.670949

[12] Chengjun Liu L , Wechsler H . Gabor feature based classification using the enhanced fisher linear discriminant model for face recognition. IEEE Trans Image Process. 2002;11(4):467‐476. 10.1109/TIP.2002.999679 18244647

[13] Ahonen T , Hadid A , Pietikainen M . Face description with local binary patterns: application to face recognition. IEEE Trans Pattern Anal Mach Intell. 2006;28(12):2037‐2041. 10.1109/TPAMI.2006.244 17108377

[14] Moon H , Phillips PJ . Computational and performance aspects of PCA‐based face‐recognition algorithms. Perception. 2001;30(3):303‐321. 10.1068/p2896 11374202

[15] Maruthapillai V , Murugappan M . Optimal geometrical set for automated marker placement to virtualized real‐time facial emotions. PLoS One. 2016;11(2):e0149003. 10.1371/journal.pone.0149003 26859884 PMC4747560

[16] Phillips PJ , White D . The state of modelling face processing in humans with deep learning. *Br J Psychol*. 2025. 10.1111/bjop.12794 PMC13051013 40364689

[17] Karnati M , Seal A , Krejcar O , et al. FER‐net: facial expression recognition using deep neural net. Neural Comput Applic. 2021;33:9125‐9136. 10.1007/s00521-020-05676-y

[18] Karnati M , Seal A , Yazidi A , Krejcar O . FLEPNet: feature level ensemble parallel network for facial expression recognition. IEEE Trans Affect Comput. 2022;13(4):2058‐2070. 10.1109/TAFFC.2022.3208309

[19] Raja SN , Carr DB , Cohen M , et al. The revised international association for the study of pain definition of pain: concepts, challenges, and compromises. Pain. 2020;161(9):1976‐1982. 10.1097/j.pain.0000000000001939 32694387 PMC7680716

[20] Park I , Park JH , Yoon J , et al. Artificial intelligence model predicting postoperative pain using facial expressions: a pilot study. J Clin Monit Comput. 2024;38(2):261‐270. 10.1007/s10877-023-01100-7 38150126

[21] Sabater‐Gárriz Á , Gaya‐Morey FX , Buades‐Rubio JM , Manresa‐Yee C , Montoya P , Riquelme I . Automated facial recognition system using deep learning for pain assessment in adults with cerebral palsy. Digit Health. 2024;10:20552076241259664. 10.1177/20552076241259664 38846372 PMC11155325

[22] Rissel C , Tate N , Moore L , et al. Assessing pain using facial recognition software among Aboriginal aged care residents with cognitive impairment: a retrospective cohort study. Australas J Ageing. 2023;42(2):317‐324. 10.1111/ajag.13170 36847297

[23] Giordano V , Luister A , Vettorazzi E , et al. Comparative analysis of artificial intelligence and expert assessments in detecting neonatal procedural pain. Sci Rep. 2024;14(1):20374. 10.1038/s41598-024-71278-6 39223215 PMC11369161

[24] Zhao Y , Zhu H , Chen X , et al. Pose‐invariant and occlusion‐robust neonatal facial pain assessment. Comput Biol Med. 2023;165:107462. 10.1016/j.compbiomed.2023.107462 37716244

[25] Wang Y , Hu F , Xu X , et al. Clinical delineation and genotype–phenotype correlation in 104 children with kabuki syndrome: a single‐center, cross‐sectional and follow‐up study in China. Eur J Pediatr. 2025;184(4):271. 10.1007/s00431-025-06103-x 40146326 PMC11950074

[26] Qiang J , Hong W , Sun Y , et al. Evaluation of an AI facial recognition system for Turner Syndrome screening and facial complexity: a prospective cohort. Int J Med Inform. 2025;203:105976. 10.1016/j.ijmedinf.2025.105976 40479775

[27] Jin B , Qu Y , Zhang L , Gao Z . Diagnosing Parkinson disease through facial expression recognition: video analysis. J Med Internet Res. 2020;22(7):e18697. 10.2196/18697 32673247 PMC7382014

[28] Ruiter AM , Wang Z , Yin Z , et al. Assessing facial weakness in myasthenia gravis with facial recognition software and deep learning. Ann Clin Transl Neurol. 2023;10(8):1314‐1325. 10.1002/acn3.51823 37292032 PMC10424649

[29] Ye J , Yu Y , Wang Q , et al. CmdVIT: a voluntary facial expression recognition model for complex mental disorders. IEEE Trans Image Process. 2025;34:3013‐3024. 10.1109/TIP.2025.3567825 40366830

[30] Selvi S , Parvathy M . Improving facial expression recognition for autism with IDenseNet‐RCAformer under occlusions. Int J Dev Neurosci. 2025;85(1):e10391. 10.1002/jdn.10391 39600258

[31] Zhang Z , Zhang T , Yang J . Facial recognition and analysis: a machine learning‐based pathway to corporate mental health management. Digit Health. 2025;11:20552076251335542. 10.1177/20552076251335542 40297378 PMC12035250

[32] Yoonesi S , Abedi Azar R , Arab Bafrani M , et al. Facial expression deep learning algorithms in the detection of neurological disorders: a systematic review and meta‐analysis. Biomed Eng Online. 2025;24(1):64. 10.1186/s12938-025-01396-3 40405223 PMC12096636

[33] Xing W , Shi Y , Wu C , Wang Y , Wang X . Predicting blood pressure from face videos using face diagnosis theory and deep neural networks technique. Comput Biol Med. 2023;164:107112. 10.1016/j.compbiomed.2023.107112 37481950

[34] Jingzhi Z , Xingzhao Z , Yao L , et al. Traditional Chinese Medicine five‐tone intelligent diagnosis and treatment system. J Tradit Chin Med. 2025;45(3):702‐710. 10.19852/j.cnki.jtcm.2025.03.021 40524310 PMC12134327

[35] Pérez‐Zuñiga G , Arce D , Gibaja S , et al. Qhali: a humanoid robot for assisting in mental health treatment. Sensors. 2024;24(4):1321. 10.3390/s24041321 38400478 PMC10891936

[36] Yan Z , Song Y , Zhou R , Wang L , Wang Z , Dai Z . Facial expression realization of humanoid robot head and strain‐based anthropomorphic evaluation of robot facial expressions. Biomimetics. 2024;9(3):122. 10.3390/biomimetics9030122 38534807 PMC10967709

[37] Dai N , Zhang K , Zhang F , et al. AI‐assisted flexible electronics in humanoid robot heads for natural and authentic facial expressions. Innovation (Camb). 2025;6(2):100752. 10.1016/j.xinn.2024.100752 39991473 PMC11846030

[38] Siddharth , Jung TP , Sejnowski TJ . Utilizing deep learning towards multi‐modal bio‐sensing and vision‐based affective computing. IEEE Trans Affect Comput. 2022;13(1):96‐107. 10.1109/TAFFC.2019.2916015

[39] Zhang Y , Cheng C , Wang S , Xia T . Emotion recognition using heterogeneous convolutional neural networks combined with multimodal factorized bilinear pooling. Biomed Signal Process Control. 2022;77:103877. 10.1016/j.bspc.2022.103877

[40] Yin Y , Zheng X , Hu B , Zhang Y , Cui X . EEG emotion recognition using fusion model of graph convolutional neural networks and LSTM. Appl Soft Comput. 2021;100:106954. 10.1016/j.asoc.2020.106954

[41] Wang S , Qu J , Zhang Y , Zhang Y . Multimodal emotion recognition from EEG signals and facial expressions. IEEE Access. 2023;11:33061‐33068. 10.1109/ACCESS.2023.3263670

[42] Liu D , Cui J , Pan Z , Zhang H , Cao J , Kong W . Machine to brain: facial expression recognition using brain machine generative adversarial networks. Cogn Neurodyn. 2024;18(3):863‐875. 10.1007/s11571-023-09946-y 38826642 PMC11143176

[43] Chandler JA , Van der Loos KI , Boehnke S , Beaudry JS , Buchman DZ , Illes J . Brain computer interfaces and communication disabilities: ethical, legal, and social aspects of decoding speech from the brain. Front Hum Neurosci. 2022;16:841035. 10.3389/fnhum.2022.841035 35529778 PMC9069963

[44] Girdhar N , Sharma D , Kumar R , Sahu M , Lin CC . Emerging trends in biomedical trait‐based human identification: a bibliometric analysis. SLAS Technol. 2024;29(3):100136. 10.1016/j.slast.2024.100136 38677477

[45] Ahmad A , Li Z , Iqbal S , et al. A comprehensive bibliometric survey of micro‐expression recognition system based on deep learning. Heliyon. 2024;10(5):e27392. 10.1016/j.heliyon.2024.e27392 38495163 PMC10943397

